# Accuracy of arch expansion with two thermoplastic materials in Invisalign^®^ patients: EX30^®^ and SmartTrack^®^

**DOI:** 10.1590/2177-6709.29.2.e2423212.oar

**Published:** 2024-06-10

**Authors:** Raquel Bueno MEDEIROS, Renata Faria SANTOS, Jose Augusto MENDES-MIGUEL, Eduardo Kant Colunga ROTHIER, Fausto Medeiros MENDES, Gladys Cristina DOMINGUEZ

**Affiliations:** 1 Universidade de São Paulo, Faculdade de Odontologia, Departamento de Ortodontia (São Paulo/SP, Brazil).; 2 Universidade Estadual do Rio de Janeiro, Faculdade de Odontologia, Departamento de Ortodontia (Rio de Janeiro/RJ, Brazil).; 3 Private practice (Rio de Janeiro, Brazil).; 4 Universidade de São Paulo, Faculdade de Odontologia, Departamento de Odontopediatria (São Paulo/SP, Brazil).

**Keywords:** Removable orthodontic appliances, Tooth movement, Retrospective studies, Malocclusion, Aparelho ortodôntico removível, Movimentação dentária, Estudos retrospectivos, Má oclusão

## Abstract

**Objective:**

The purpose of this retrospective study was to compare accuracy of arch expansion using two different thermoplastic materials in Invisalign aligners: EX30® (Polyethylene Terephthalate Glycol, or PETG) and SmartTrack® (polyurethane).

**Methods:**

The study sample comprised 65 adult patients consecutively treated with Invisalign from two private practices: group 1 - treated with EX30® (358 teeth) and group 2 - treated with SmartTrack® (888 teeth). Six hundred and twenty-three measurements were assessed in three digital models throughout treatment: model 1 - initial, model 2 - predicted tooth position, and model 3 - achieved position. Sixteen reference points per arch were marked and, after best alignment, 2 points per tooth were copied from one digital model to another. Linear values of both arches were measured for canines, premolars, and first molars: on lingual gingival margins and cusp tips of every tooth. Comparisons were performed by Wilcoxon and Mann-Whitney test.

**Results:**

Both termoplastic materials presented significant differences between predicted and achieved values for all measurements, except for the lower molar cusp tip in the SmartTrack® group. There is no statistical difference in the accuracy of transverse expansion between these two materials. Overall accuracy for EX30® aligners in maxilla and mandible were found to be 37 and 38%, respectively; and Smarttrack® presented an overall accuracy of 56.62% in the maxilla and 68.72% in the mandible.

**Conclusions:**

It is not possible to affirm one material expands better than the other. Further controlled clinical studies should be conducted comparing SmartTrack® and EX30® under similar conditions.

## RESUMO

**Objetivo:**

O objetivo deste estudo retrospectivo foi comparar a acurácia na expansão da arcada utilizando alinhadores Invisalign com dois tipos de material termoplástico: EX30^®^ (Polietileno Tereftalato Glicol, ou PETG) e SmartTrack^®^ (poliuretano).

**Método:**

A amostra desse estudo incluiu 65 pacientes adultos tratados consecutivamente em dois consultórios particulares: grupo 1 - tratado com EX30^®^ (358 dentes), e grupo 2 - tratado com SmartTrack^®^ (888 dentes). Foram avaliadas 623 medidas em três pares de modelos digitais, ao longo do tratamento: modelo 1 - inicial, modelo 2 - posição dentária planejada, modelo 3 - posição alcançada. Foram marcados 16 pontos de referência por arcada e, após o *bestfit*, 2 pontos por arcada foram copiados de um modelo digital para o outro. Medidas lineares de ambas as arcadas foram aferidas para caninos, pré-molares e primeiros molares, localizadas na margem gengival lingual e ponta de cúspide de todos os dentes. Foram realizadas comparações usando os testes Wilcoxon e Mann-Whitney.

**Resultados:**

Ambos os materiais termoplásticos apresentaram diferenças significativas entre os valores planejados e alcançados em todas as medidas, exceto na ponta de cúspide dos primeiros molares inferiores do grupo SmartTrack^®^. Não houve diferença estatística entre esses dois materiais quanto à acurácia da expansão transversa. Em termos gerais, a acurácia dos alinhadores EX30^®^ na maxila e mandíbula foi de 37% e 38%, respectivamente. O grupo SmartTrack^®^ apresentou acurácia de 56,62% e 68,72% na maxila e mandíbula, respectivamente.

**Conclusões:**

Não foi possível afirmar que um material expande melhor que o outro. Estudos clínicos controlados futuros devem ser conduzidos comparando SmartTrack^®^ e EX30^®^ sob condições similares.

## INTRODUCTION

Since its launch in 1999, Invisalign^®^ has grown rapidly in a worldwide consumer demand as an esthethic alternative to fixed appliances. The Invisalign^®^ system operates a CAD/CAM technology to help technicians and dentists elaborate a treatment plan that relies on individualized aligners designed to move teeth in 14 days intervals.^1^

Over the last decade, there was a shift on the complexity of malocclusions being treated by Invisalign, the system evolved from treating mild dental crowding to borderline cases.^2^

Nevertheless, dental crowding is still one of the main reasons people seek orthodontic treatment. Expansion of a constricted arch may resolve dental crowding by increasing the arch length, and it is also an alternative to avoid interproximal reduction.^3^ ^-^ ^6^

Aligners currently on the market may have a similar external appearance, but in reality they differ in many aspects, e.g. manufacturing material, thickness, and clinical protocol.^7^ ^-^ ^9^ In an ideal world, clear aligners should be able to exert light constant forces to promote physiological tooth movement; however, studies on clear aligner behavior under stress have reported forces that are not constant and may differ considerably overtime.^9^ The magnitude of the force applied and the properties of the material used for aligner manufacture tend to have a direct effect on performance.^10^ Lombardo et al.^7^ study results confirm that aligners on the market will perform differently depending on their thickness and construction material.

In 2013, there was a worldwide change in the construction material of Invisalign^®^, when SmartTrack^®^ (polyurethane) aligners were announced as a superior option, with better tooth movement accuracy, higher flexibility, and more patient comfort. From that moment on, the former EX30^®^ (Polyethylene Terephthalate Glycol, or PETG) aligners were replaced by SmartTrack^®^. However, that shift from PETG to polyurethane material was not observed in other aligner companies, since most of them currently rely on modified PETG, polypropylene and polycarbonate.^7^ ^-^ ^10^

Until this moment, the majority of scientific data regarding tooth movement accuracy with clear aligners does not consider aligner material a relevant variable.^11^ ^-^ ^14^ And there is only a limited number of studies on tooth movement accuracy comparing different plastic materials, specially in transverse expansion. The hypothetical superiority of polyurethane over other aligner materials is an assumption that has prevailed over the years, nonetheless further studies are still needed to elucidate the matter. Thus, the objective of the present study was to assess the accuracy of maxillary and mandibular expansion planned by ClinCheck^®^ software for the use of EX30^®^ and SmartTrack^®^ aligners at the end of the first treatment phase. 

## MATERIAL AND METHODS

The Ethics Committee of University of São Paulo School of Dentistry approved this retrospective study (2.865.423) on January 2019. 

Sample size calculation was based on the linear distance between two teeth, with an effect size of 0.88, α error probability of 0.05, and power of 80%. It was estimated to be 13 linear measurements per group. The sample was obtained from two private practices, both with a high degree of expertise with Invisalign^®^. 

Patient selection was based on the following inclusion criteria: age between 22 and 55 years, non extraction treatment, no missing teeth, no midcourse corrections, no combined treatment with fixed appliances or any other auxiliary appliance. Exclusion criteria were: presence of autoimmune disease, long-term use of medication three months prior to the beginning of treatment (non-steroidal anti-inflammatory, cortisone, immune suppressive, and bisphosphonate drugs), pregnant and/or lactating women, and final digital scans should not exceed 45 days post-treatment.

According to the eligibility criteria, the principal investigator remotely recruited digital files (.stl) via ClinCheck^®^ from 74 patients over a 30 day period (Sept. 2018). After consulting physical archives, 5 patients treated with auxiliary mechanics and 2 with midcourse correction were excluded from the study. Two other patients presenting corrupted files were also excluded. Patient data were de-identified, and a unique number was assigned for each patient.

The final sample consisted of 65 consecutive patients divided into two groups: EX30^®^ and SmartTrack^®^. EX30^®^ group included 22 patients (9 male and 13 female) with mean age of 37 years (min = 22 years; max = 51 years) and mean treatment time of 8,8 months (min = 5 months; max = 13 months). SmartTrack^®^ group included 43 patients (12 male and 31 female) with mean age of 35 years (min = 23 years; max = 55 years) and mean treatment time of 12,8 months (min = 7 months; max = 25,5 months). Patients had to change aligners every two weeks. 

The sample was classified according to the manufacturing material used for aligners: EX30^®^ group (Polyethylene Terephthalate Glycol, or PETG) (n = 179 linear measurements) - aligners manufactured until January 27^th^ 2013; SmartTrack^®^ group (polyurethane) (n = 444 linear measurements) - aligners manufactured from January 28^th^ 2013 until the present date.

**Figure 1:**
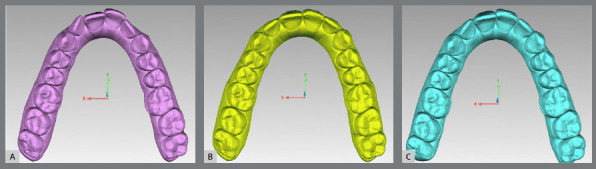
Model 1 (A), Model 2 (B) and Model 3 (C).

For each patient, three sets of digital files (.stl) were uploaded into Geomagic Control^®^ software (North Carolina, USA): Model 1 - before treatment, Model 2 - expansion predicted by ClinCheck^®^, and Model 3 - expansion achieved at the end of the first treatment phase (Fig 1). 

**Figure 2:**
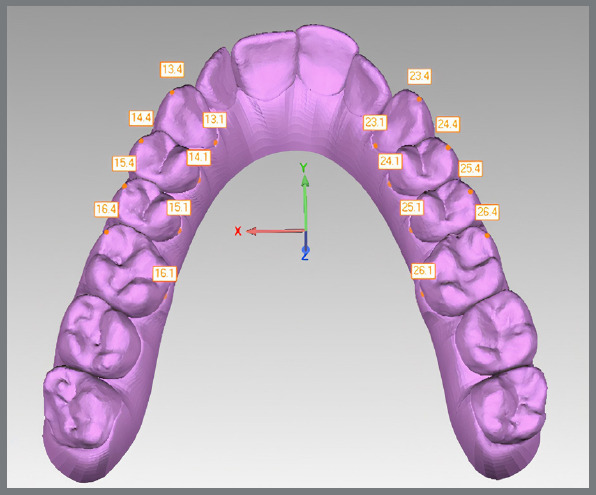
Sixteen reference points were manually marked on model 1 at the cusp tip and gingival margin regions of canines, first and second premolars, and first molars.

**Figure 3:**
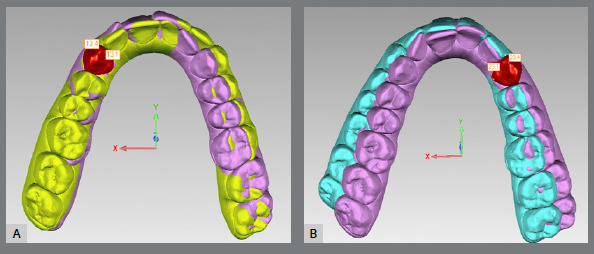
The teeth in red indicates best fit alignment of the upper right canines of models 1 and 2 (A), and models 1 and 3 (B).

**Figure 4:**
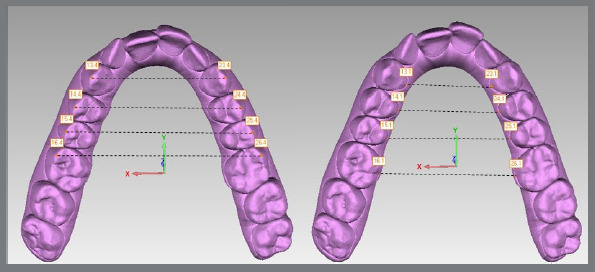
Linear values of the upper arch were obtained from X axis, measured at the cusp tip and lingual points at the gingival margin of canines, first and second premolars, and first molars.

Sixteen reference points per arch were marked in Model 1 (Fig 2) and, after bestfit alignment, 2 points per tooth were copied from Model 1 to Model 2, and from Model 1 to Model 3 (Fig 3). In case of wear facets, the tooth was excluded, because bestfit alignment could not be performed. Arch width measurements were recorded using Geomagic Control’s digital caliper in the three sets of digital models. Linear values of upper and lower arch widths were measured at the cusp tip and lingual points at the gingival margin of canines, first and second premolars, and first molars (Fig 4). Linear measurements were obtained from X axis only, additional values from Y and Z axis were discarded. Predicted tooth movement under 0.5 mm were not considered for analysis. The second molars were not evaluated in this study.

The following transverse linear values were measured for each model:

» Intercanine width tip: linear distance between cusp tips of the canines.
» Intercanine width gingival: linear distance between lingual point of the gingival margin of canines.
» First premolar width tip: linear distance between the buccal cusp tips of first premolars.
» First premolar width gingival: linear distance between the lingual point of the gingival margin of first premolars.
» Second premolar width tip: linear distance between the buccal cusp tips of second premolars.
» Second premolar width gingival: linear distance between the lingual point of the gingival margin of second premolars.
» First molar width tip: linear distance between the mesiobuccal cusp tip of the first molars.
» First molar width gingival: linear distance between the lingual point of the gingival margin of the first molars.

The accuracy of arch expansion was assessed by comparing the discrepancies of predicted tooth position idealized by ClinCheck^®^ (Model 2 - Model 1) and the achieved tooth positions obtained at the end of the first treatment phase (Model 3 - Model 1). The formula applied in this study to measure the percentage accuracy for arch expansion was based on the one proposed by Houle et al^3^: *Percentage accuracy = [(achieved post-treatment distance - pretreatment distance / predicted post-treatment distance - pretreatment distance) x 100%].*

This study did not assess any data of anterior teeth or angular movements, such as rotation, inclination and torque. The analysis focused on linear tooth movement of posterior teeth. To reduce potential source of bias, patients records were de-identified, and all data extraction was performed by the principal investigator, while all statistical methods were handled by an assistant. 

## STATISTICAL ANALYSIS

Statistical analysis was performed using SPSS v. 25 (Statistical Package for the Social Sciences, IBM Corp, Chicago, USA). Outcome variables were: predicted expansion (predicted posttreatment distance - pretreatment distance) and achieved expansion (achieved posttreatment distance - pretreatment distance). Variables were submitted to Shapiro-Wilk and to Levene’s test, to assess normality and homogeneity of variance of these variables. They were expressed as median (1^st^ and 3^rd^ quartiles), minimum and maximum values. Comparisons between predicted and achieved expansions were performed by Wilcoxon test. Comparisons between the accuracy of EX30^®^ and SmartTrack^®^ expansions were performed by Mann-Whitney test. Values of *p* ˂ 0.05 were considered statistically significant.

For the inter-rate reliability, 98 measurements (15%) were randomly selected and were obtained again after three weeks. The ICC (2-way mixed, single measurement, absolute agreement) was used for analyses. 

## RESULTS

Assessed teeth presented a wide range of predicted tooth movement (min = 0.50mm; max = 11.59mm), any predicted tooth movement under 0.5 mm was not considered for analysis. Data of achieved tooth movement ranged from negative to positive values (min = -1.19mm; max = 6.75mm).

All measurements present a statistical difference between predicted and achieved outcomes in patients treated with EX30^®^ and SmartTrack^®^ aligners, except lower molar cusp tip in the SmartTrack^®^ group (*p*=0.062).

The most reliable areas to predict transverse changes with EX30^®^ aligners were the first premolar tip in the maxilla and the canine tip in the mandible, with 55.73% and 69.91% of achieved changes, respectively. The most reliable areas with SmartTrack^®^ aligners were the second premolar tip in the maxilla and first molar tip in the mandible, with 76% and 83.32% of achieved changes, respectively.

Table 1 demonstrates the first premolar tip as the most accurate area with EX30^®^ aligners in the maxilla, in which the median predicted change was 1.78 mm (min = 0.5mm; max = 5.56mm), and at the end of treatment the real achieved change was 0.59 mm (min = -0.14mm; max = 3.18mm). In that case, EX30^®^ aligners have reached up to one third of what it was initially planned. Table 2 demonstrates the canine tip as the most accurate area with EX30^®^ aligners in the mandible, reaching approximately half of what it was predicted on ClinCheck^®^, the median predicted change was 1.71 mm (min = 0.70mm; max = 3.33mm), and the real achieved change was 0.85 mm (min = -0.25mm; max = 2.82mm). Prediction accuracy with EX30^®^ aligners ranged from 14.38% (lower first molar gingival) to 69.91% (lower canine tip).

**Table 1:** Accuracy of tooth movement ( TM ) changes with EX30^®^ aligners in the maxilla.

Tooth type	n	Predicted TM* Median (1st; 3rd quartiles)	Min-Max	Achieved TM* Median (1st; 3rd quartiles)	Min-Max	Difference (Predicted - Achieved) Median (1st; 3rd quartiles)	P value**	Accuracy of change
Canine tip	12	1.87 (0.78; 4.43)	0.62-5.41	0.53 (0.05; 1.33)	-0.30-4.90	0.74 (0.37; 3.08)	0.005	38.29%
Canine gingival	13	2.65 (0.96; 3.47)	0.52-3.88	0.46 (-0.01; 0.98)	-0.26-3.17	1.64 (0.66; 2.50)	0.002	25.16%
First premolar tip	16	1.78 (0.79; 2.94)	0.50-5.56	0.59 (0.23; 1.22)	-0.14-3.18	0.66 (0.11; 1.82)	0.005	55.73%
First premolar gingival	14	1.83 (1.00; 2.84)	0.58-3.28	0.52 (0.12; 0.92)	-0.34-1.74	1.13 (0.49; 2.19)	0.001	33.72%
Second premolar tip	14	1.86 (0.99; 3.19)	0.61-5.00	0.77 (0.15; 1.35)	-0.23-4.97	1.03 (0.42; 1.67)	0.002	49.06%
Second premolar gingival	10	1.62 (1.25; 2.83)	0.68-4.14	0.61 (-0.01; 1.15)	-0.06-3.84	1.21 (0.41; 1.73)	0.005	35.60%
First molar tip	9	2.01 (0.96; 2.95)	0.59-5.76	0.31 (0.18; 1.48)	-0.16-5.64	0.89 (0.49; 1.97)	0.008	26.75%
First molar gingival	9	1.70 (1.04; 3.02)	0.61-4.10	0.46 (0.25; 1.33)	0.11-3.57	0.75 (0.51; 1.53)	0.008	42.26%

Comparison between predicted and achieved expansion was performed by Wilcoxon Test. * Median values in millimeters; ** p < 0.05.

**Table 2:** Accuracy of tooth movement ( TM ) changes with EX30^®^ aligners in the mandible.

Tooth type	n	Predicted TM* Median (1st; 3rd quartiles)	Min-Max	Achieved TM* Median (1st; 3rd quartiles)	Min-Max	Difference (Predicted - Achieved) Median (1st; 3rd quartiles)	P value**	Accuracy of change
Canine tip	11	1.71 (0.87; 2.49)	0.70-3.33	0.85 (0.13; 1.75)	-0.25-2.82	0.26 (0.00; 1.49)	0.06	69.91%
Canine gingival	17	1.40 (0.83; 2.37)	0.54-4.0	0.48 (-0.01; 1.21)	-0.11-1.63	0.75 (0.20; 1.75)	0.001	35.47%
First premolar tip	14	1.48 (0.93; 2.38)	0.68-4.68	0.32 (0.02; 0.80)	-0.10-1.40	0.82 (0.05; 2.31)	0.001	16.26%
First premolar gingival	9	1.79 (0.94; 2.60)	0.90-3.22	0.17 (-0.01; 0.74)	-0.27-1.45	1.12 (0.40; 2.66)	0.008	19.41%
Second premolar tip	8	2.62 (1.13; 3.52)	0.82-3.70	1.22 (0.56; 1.92)	0.46-2.18	1.26 (0.40; 1.62)	0.017	52.96%
Second premolar gingival	10	1.06 (0.78; 1.75)	0.71-2.48	0.37 (0.22; 1.00)	0.10-1.22	0.72 (0.50; 1.33)	0.007	34.76 %
First molar tip	7	1.56 (0.61; 2.07)	0.50-2.32	0.53 (0.08; 0.85)	0.05-1.31	0.90 (0.03; 1.69)	0.043	42.26 %
First molar gingival	6	1.09 (0.88; 1.36)	0.51-1.92	0.16 (0.01;0.63)	-0.001-1.12	0.95 (0.37; 1.16)	0.046	14.38 %

Comparison between predicted and achieved expansion was performed by Wilcoxon test. *Median values in millimeters; ** p < 0.05.

Table 3 demonstrates the second premolar tip as the most accurate area with Smarttrack^®^ aligners in the maxilla: the median predicted change was 2.0 mm (min = 0.54mm; max = 2.82mm), and at the end of treatment the real change achieved was 1.57mm (min = -1.17mm; max = 5.86mm). Table 4 demonstrates the first molar tip as the most accurate area with Smarttrack^®^ aligners in the mandible: the median predicted change was 1.16mm (min = 0.50mm; max = 7.45mm), and the real change achieved was 1.18mm (min = 0.0mm; max = 5.35mm). Prediction accuracy with SmartTrack^®^ aligners ranged from 40.80% (lower canine gingival) to 83.32% (lower first molar tip).

**Table 3:** Accuracy of tooth movement ( TM ) changes with SmartTrack^®^ aligners in the maxilla.

Tooth type	n	Predicted TM* Median (1st; 3rd quartiles)	Min-Max	Achieved TM* Median (1st; 3rd quartiles)	Min-Max	Difference (Predicted - Achieved) Median (1st; 3rd quartiles)	P value**	Accuracy of change
Canine tip	26	1.45 (0.96; 2.42)	0.50- 4.12	1.08 (0.46; 1.35)	-0.53- 2.43	0.60 (0.11; 1.03)	<0.001	63.19%
Canine gingival	28	1.77 (1.03; 2.67)	0.58- 9.65	0.68 (0.29; 1.24)	-0.88- 2.01	1.03 (0.46; 1.51)	<0.001	46.54%
First premolar tip	32	2.04 (1.33; 3.49)	0.50- 7.91	1.37 (0.48; 2.28)	-0.21- 5.83	0.60 (0.11; 1.58)	<0.001	66.10%
First premolar gingival	29	2.02 (1.34; 2.73)	0.73- 4.81	1.08 (0.53; 1.94)	-0.33- 3.50	0.99 (0.41; 1.73)	<0.001	56.30%
Second premolar tip	30	2.00 (1.31; 3.61)	0.54- 7.41	1.57 (0.80; 1.98)	-1.17- 5.86	0.47 (0.04; 1.67)	<0.001	76.00%
Second premolar gingival	27	1.72 (1.37; 2.65)	0.50- 5.18	1.14 (0.37; 1.38)	-0.72- 3.96	0.77 (0.39; 1.45)	<0.001	53.57%
First molar tip	25	1.43 (0.92; 2.84)	0.51- 4.66	0.68 (0.25; 1.41)	-0.81- 3.50	0.63 (0.10; 2.16)	<0.001	43.95%
First molar gingival	23	1.05 (0.77; 1.65)	0.51- 4.29	0.62 (1.76; 0.98)	-1.19- 2.08	0.49 (0.15; 1.06)	<0.001	57.28%

Comparison between predicted and achieved expansion was performed by Wilcoxon test. *Median values in millimeters; ** p < 0.05.

The least reliable areas to predict transverse changes with EX30^®^ aligners were the lingual gingival margin of canine in the maxilla and the lingual gingival margin first molar in the mandible, with 25.16% and 14.38% of achieved changes, respectively. The least reliable areas to predict changes with Smarttrack^®^ aligners were the first molar tip in the maxilla and the canine gingival in the mandible, with 43.95% and 40.80% of achieved changes, respectively.

Table 1 demonstrates lingual gingival margin of the upper canines as the least accurate area with EX30^®^ aligners: the median predicted change was 2.65mm (min = 0.52mm; max = 3.88mm), and at the end of treatment the real change achieved was 0.46mm (min = -0.26mm; max = 3.17mm). Table 2 demonstrates lingual gingival margin of the first molar as the least accurate with EX30^®^ aligners: the median predicted change was 1.09 mm (min = 0.51mm; max = 1.92mm), and at the end of treatment the real change achieved was 0.16mm (min = -0.001mm; max = 1.12mm). 

Table 3 demonstrates the first molar tip as the least accurate with SmartTrack^®^ aligners in the maxilla: the median predicted change was 1.43mm (min = 0.51mm; max = 4.66mm), and the real change achieved was 0.68mm (min = -0.081mm; max = 3.50mm). Table 4 demonstrates the lingual gingival margin of the canines as the least accurate with SmartTrack^®^ aligners in the mandible: the median predicted change was 1.69mm (min = 0.50mm; max = 4.96mm), and the real change achieved was 0.67mm (min = -0.43mm; max = 3.28mm).

**Table 4:** Accuracy of tooth movement ( TM ) changes with SmartTrack^®^ aligners in the mandible.

Tooth type	n	Predicted TM* Median (1st; 3rd quartiles)	Min-Max	Achieved TM* Median (1st; 3rd quartiles)	Min-Max	Difference (Predicted - Achieved) Median (1st; 3rd quartiles)	P value**	Accuracy of change
Canine tip	28	1.74 (1.01; 2.52)	0.62- 6.71	0.89 (0.35; 1.99)	-0.34- 3.85	0.49 (0.18; 0.80)	<0.001	72.71%
Canine gingival	36	1.69 (0.99; 2.91)	0.50- 4.96	0.67 (0.12; 1.29)	-0.43- 3.28	0.77 (0.39; 1.73)	<0.001	40.80%
First premolar tip	25	2.21 (1.43; 3.61)	0.68- 8.69	1.22 (0.69; 2.53)	0.04- 6.44	0.41 (0.00; 1.49)	0.005	76.66%
First premolar gingival	26	1.44 (0.80; 2.37)	0.50- 5.73	0.83 (0.45; 1.53)	-0.56- 4.60	0.38 (-0.03; 1.14)	0.003	70.40%
Second premolar tip	36	2.31 (0.93; 4.10)	0.55- 11.59	1.68 (0.65; 2.43)	-0.05- 6.75	0.46 (-0.13; 1.76)	<0.001	64.12%
Second premolar gingival	26	1.41 (0.92; 2.48)	0.50- 7.41	1.01 (0.41; 1.58)	-0.19- 4.60	0.40 (-0.11; 1.11)	0.020	73.10%
First molar tip	26	1.16 (0.78; 2.96)	0.50- 7.45	1.18 (0.75; 1.76)	0.00- 5.35	0.34 (-0.22; 0.85)	0.062	83.32%
First molar gingival	21	0.94 (0.73; 3.04)	0.57- 5.07	0.82 (0.40; 1.11)	-0.23- 3.31	0.34 (0.06; 1.14)	0.004	67.88%

Comparison between predicted and achieved expansion was performed by Wilcoxon test. *Median values in millimeters; ** p < 0.05.

Tables 5 and 6 provide a comparison of arch expansion accuracy obtained from EX30^®^ versus SmartTrack^®^ aligners in the maxilla and mandible, respectively. There was no statistical difference in the accuracy of transverse expansion between these two materials (*p* ˃ 0.05).

**Table 5:** Comparison of arch expansion accuracy obtained from EX30^®^ versus SmartTrack^®^ in the maxilla.

Tooth type	EX30 group (n)	EX30 Difference* (Predicted - Achieved) Median (1st; 3rd quartiles)	Min-Max	SmartTrack group (n)	SmartTrack Difference* (Predicted - Achieved) Median (1st; 3rd quartiles)	Min-Max	P value**
Canine tip	12	0.74 (0.37; 3.08)	(-0.27-5.41)	26	0.60 (0.11; 1.03)	(-0.26-3.08)	0.72
Canine gingival	13	1.64 (0.66; 2.50)	(-0.35-3.11)	28	1.03 (0.46; 1.51)	(-0.26-10.54)	0.43
First premolar tip	16	0.66 (0.11; 1.82)	(-0.47-5.33)	32	0.60 (0.12; 1.58)	(-0.26-10.54)	0.75
First premolar gingival	14	1.13 (0.49; 2.19)	(-0.01-3.29)	29	0.99 (0.41; 1.73)	(-0.73-3.35)	0.82
Second premolar tip	14	1.03 (0.42; 1.67)	(-0.29-3.28)	30	0.47 (0.04; 1.67)	(-0.42-4.34)	0.33
Second premolar gingival	10	1.21 (0.41; 1.73)	(0.6-2.32)	27	0.77 (0.39; 1.45)	(-0.17-4.12)	0.63
First molar tip	9	0.89 (0.49; 1.97)	(0.12-2.10)	25	0.63 (0.10; 1.16)	(-0.78-4.53)	0.43
First molar gingival	9	0.75 (0.51; 1.53)	(0.35-2.54)	23	0.49 (0.15; 1.12)	(-0.90-4.40)	0.43

Comparison between EX30^®^ and SmartTrack^®^ differences was performed by Mann-Whitney test. *Median values in millimeters; ** p < 0.05.

**Table 6:** Comparison of arch expansion accuracy obtained from EX30^®^ versus SmartTrack^®^ in the mandible.

Tooth type	EX30 group (n)	EX30 Difference* (Predicted - Achieved) Median (1st; 3rd quartiles)	Min-Max	SmartTrack Group (n)	SmartTrack Difference* (Predicted - Achieved) Median (1st; 3rd quartiles)	Min-Max	P value**
Canine tip	11	0.26 (0.01; 1.49)	(-1.10-3.60)	28	0.49 (0.18; 0.80)	(-0.76-6.86)	0.92
Canine gingival	17	0.75 (0.20; 1.75)	(-0.13-2.89)	36	0.77 (0.39; 1.73)	(-0.38-4.32)	0.92
First premolar tip	14	0.82 (0.51; 2.31)	(-0.21-4.65)	25	0.41 (0.00; 1.49)	(-1.40-7.71)	0.26
First premolar gingival	9	1.12 (0.40; 2.66)	(0.21-3.22)	26	0.38 (-0.03; 1.14)	(-0.58-5.07)	0.38
Second premolar tip	8	1.26 (0.40; 1.61)	(-0.29-2.68)	36	0.46 (-0.13; 1.76)	(-0.89-9.75)	0.69
Second premolar gingival	10	0.72 (0.50; 1.33)	(-0.43-1.45)	26	0.40 (-0.11; 1.11)	(-2.23-6.68)	0.26
First molar tip	7	0.90 (0.03; 1.69)	(-0.06-1.85)	26	0.34 (-0.22; 0.85)	(-1.51-5.94)	0.34
First molar gingival	6	0.95 (0.37; 1.16)	(-0.05-1.45)	21	0.34 (0.06; 1.14)	(-0.83-4.91)	0.077

Comparison between EX30 and SmartTrack differences was performed by Mann-Whitney test. *Median values in millimeters; ** p < 0.05.

## DISCUSSION

The goal of this study was to assess accuracy of transverse expansion of two different plastic materials used for Invisalign^®^ aligners: EX30^®^ and SmartTrack^®^. Even though Invisalign^®^ stopped producing EX30^®^ aligners in 2013, PETG is still one of the most used plastics for aligner manufacturing.^7^ ^-^ ^10^

The outcomes of this study support there was no statistical difference in the accuracy of transverse expansion between EX30^®^ and SmartTrack^®^ (*p* ˃ 0.05) (Tables 5 and 6), therefore a hypothetical superiority of SmartTrack^®^ in terms of transverse expansion accuracy is not sustained by the present findings. 

The results have shown a statistical difference between predicted and achieved outcomes in all patients treated with EX30^®^ and SmartTrack^®^ aligners, except lower molar cusp tip in the SmartTrack^®^ group (*p*=0.062). A study outcome can be statistically significant, but not clinically relevant, and vice versa.^15^ Nevertheless, when predicted tooth movements are under 60% of achieved changes, it can not be overlooked or interpreted as clinically irrelevant - in such cases, clinical results are most likely unsatisfactory. Prediction accuracy above 60% has been achieved in 1/16 of the measurements with EX30^®^ aligners and in 4/16 with SmartTrack^®^ aligners (Tables 1-4). 

Overall accuracy of the upper and lower arch in the EX30^®^ group was 37% and 38%, respectively. These poor results are in accordance with prospective clinical trials performed by Kravitz et al^1^ and Solano-Mendoza et al.^4^ SmartTrack^®^ group presented higher overall accuracy rates of 56.62% and 68.72% for the upper and lower arches, respectively. Lione et al^6^ reported poor predictability for transverse expansion with SmartTrack^®^ aligners from a prospective clinical trial, and two other retrospective studies^16^ ^,^ ^17^ affirmed SmartTrack^®^ aligners are an effective tool for producing arch expansion, showing reasonable to high degree of accuracy. There is still no consensus about the accuracy of transverse expansion with SmartTrack^®^ aligners. In our study, we observed an improvement in transverse expansion accuracy rates, where poor results from EX30^®^ aligners were followed by reasonable ones obtained from SmartTrack^®^ aligners. It is clear Align^®^ Technology has evolved greatly over the years. 

One limitation of this study is the small sample size of the EX30 group. The amount of expansion requested for molars in this study was overall small, and any expansion under 0.5 mm was not considered for analysis - these two factors combined contributed to a shortage of bimolar linear measurements. Another limitation include large variation on attachment design, tooth movement sequencing according to orthodontist’s preference and G8 enhancements. This study relies on clinical decisions by two orthodontic providers.

According to Invisalign^®^, SmartTrack^®^ material would in theory improve control and predictability of tooth movements, due to its flexibility. SmartTrack^®^ has been favorably rated in a patient survey as an aligner material that provides more comfort during use, and a significant reduction in pain intensity.^18^ The latest improvements in transverse expansion accuracy are credited to the SmartTrack^®^ aligner material, which in our opinion is an erroneous interpretation of the available evidence,^16^ ^,^ ^17^ as it ignores the new protocols of G8 expansion features^19^ and restrains the exploitation of potential benefits from other aligner materials. As reported by Moshiri et al^19^, Align^®^ Technology has released new improvements for posterior arch expansion with the threshold of 0.5 mm buccal expansion to activate G8 expansion features, which include: balanced posterior forces, automatic placement of buccal root torque to avoid tipping, and optimized support attachments on the premolars and first molars. The hierarchy for tooth activation has been updated and now expansion is considered a priority to the software. Therefore, a comparison among two different aligner materials to assess transverse accuracy should be regarded with caution, due a limitation inherent to the sample. To the best of our knowledge, there is no scientific evidence that supports SmartTrack^®^ bodily expands better than EX30^®^, and for that purpose, future controlled clinical trials are needed to assess transverse expansion accuracy of the two materials under similar conditions. 

Several *in-vitro* studies on chemical and mechanical properties of the most common PETG and polyurethane (PU) based orthodontic aligners have been published over the last years,^7^ ^-^ ^10^ ^,^ ^20^ and their conclusion is unanimous that the materials tested showed significant differences in their chemical and mechanical characteristics and, therefore, differences in their clinical behavior are expected. The clinical effectiveness of PU in comparison to PETG needs to be investigated under similar conditions.

The premise that clear aligners tend to decrease its accuracy as it moves posteriorly is in accordance to our findings in the EX30^®^ group.^3^ ^,^ ^5^ ^,^ ^17^ And in contradiction to our findings for SmartTrack^®^ aligners, our data has shown an improvement in the accuracy of posterior teeth, with first molars presenting from 67.88 to 83.32% of predicted changes. A potential explanation for this finding is the overall small quantity of expansion requested for molars in this study, the increase of requested movement has a negative impact on accuracy^1^ and, theoretically minor expansions would achieve more accurate results.

Two reference points were selected to represent transverse tipping and bodily movement, i.e. cusp tip and gingival margin, respectively. The overall transverse changes in the maxilla with EX30^®^ aligners were found to be 41.46% at the cusp tips and 33.39% at the gingival margins; and in the mandible, 54.15% at the cusp tips and 24.45% at the gingival margins. SmartTrack^®^ also confirmed the tendency to incline during arch expansion, overall accuracy in the maxilla was 65.20% at the cusp tips and 52.69% at the gingival margin; and in the mandible, it was 76.16% at the cusp tip and 63.29% at the gingival margin. As reported in previous studies,^3^ ^,^ ^5^ ^,^ ^17^ ^,^ ^21^ data suggest that during dentoalveolar expansion, Invisalign predicts more bodily movement than it can actually achieve. 

Our methodology was automated and able to quantify the difference between estimated and achieved expansion obtained at the end of the first phase of treatment. The best alignment feature from Geomagic Control allows to optimally match a set of points to a CAD curve or surface, in other words, the reference points set on one tooth are precisely copied to its counterpart, eliminating the risk of disagreement between measurements when manual marking is performed.

Our findings sustain that overcorrection is needed for all cases of transverse expansion in both types of aligner material, and buccal crown torque of posterior teeth is recommended to minimize tipping. These findings are in accordance with previous studies.^5^ ^,^ ^6^ ^,^ ^16^

Future randomized clinical trials should be able to identify and explore the potential of the most common PETG and PU aligner material for the different types of tooth movement. Dental tip, rotation, extrusion, intrusion, and expansion are distinct tooth movements that may hipotethically benefit from specific chemico-physical properties of aligner materials available in the market.

## CONCLUSIONS

» The overall mean expansion accuracy has improved with SmartTrack^®^, nevertheless our findings support there was no statistical difference in the accuracy of transverse expansion between EX30^®^ and SmartTrack^®^.
» It is not possible to affirm one material expands better than the other.
» Further controlled clinical studies should be conducted comparing SmartTrack^®^ and EX30^®^ under similar conditions.
» Overcorrection and buccal crown torque are recommended for both materials to achieve planned expansion.
